# Online assessment of suicide stigma, literacy and effect in Australia’s rural farming community

**DOI:** 10.1186/s12889-018-5750-9

**Published:** 2018-07-06

**Authors:** Alison J. Kennedy, Susan A. Brumby, Vincent Lawrence Versace, Tristan Brumby-Rendell

**Affiliations:** 1 Deakin University, National Centre for Farmer Health, 75 Pigdons Road, Geelong, VIC 3216 Australia; 2 Deakin University, Deakin Rural Health, 75 Pigdons Road, Geelong, VIC 3216 Australia; 3 National Centre for Farmer Health, Western District Health Service, PO Box 283, Hamilton, VIC 3300 Australia

**Keywords:** Suicide stigma, Suicide literacy, Suicide effect, Australia, Farmer health, Rural health, Mixed method research, Digital intervention

## Abstract

**Background:**

In Australia, farming populations have been identified as having higher rates of suicide, in comparison to metropolitan, rural and regional communities. The reasons for this are unclear although stigma is considered a risk factor. This study was designed to understand the role of suicide stigma and suicide literacy and the relationship between these.

**Methods:**

A mixed-methods online intervention was developed. This paper reports on baseline quantitative data (suicide stigma, suicide literacy and suicide effect) collected from male and female rural Australian participants (*N* = 536) with an experience of suicide.

**Results:**

When compared with previous Australian community samples, our sample demonstrated higher levels of stigma and higher levels of suicide literacy. Males were more likely to have considered suicide than females. Females were more likely than males to report a devastating and ongoing effect of suicide bereavement, but less likely than a previous Australian community sample.

**Conclusion:**

Results of this study reiterate the need for improved understanding of the risk factors and experience of suicide within the context of life and work in rural Australian farming communities and how ‘best practice’ can be adapted to improve stigma reduction and suicide prevention efforts.

**Trial registration:**

This research project was registered with the Australian New Zealand Clinical Trials Registry (ANZCTR) (ACTRN12616000289415) on 7th March, 2016.

## Background

### Understanding suicide and its effect in rural farming communities

Elevated rates of rural suicide are consistently reported across developed and developing nations [1]. In Australia, farming populations have been identified as having higher rates of suicide, in comparison to metropolitan, rural and regional communities [2–6]. These suicides in farming communities occur in the absence of higher rates of diagnosed mental health conditions [7, 8], indicating that other influencing factors are at play. The reasons for suicide in farming communities are complex and intertwined [9], with a range of individual, cultural, social, geographical, financial, occupational and environmental factors contributing to suicide risk [9–12]. An increasing body of evidence identifies stigma as a risk factor for suicide [13]. This paper describes baseline data from the Australian rural farming community—including demographics and suicide experience—and levels of self-and perceived-suicide stigma, suicide literacy and suicide effect relative to previously measured community samples.

### Suicide stigma

Stigma has been variously defined as “a mark of disgrace; a stain, as on one’s reputation” [14] and “a set of negative and often unfair beliefs that a society or group of people have about something” [15]. Stigma reduces the prospect of recovery from poor mental health and complicates access to available care and resources—stigma makes people sick [16]. Stigma can be demonstrated and experienced in a number of ways [17]. Self-stigma is the experience of negative beliefs towards one self; personal stigma is negative attitudes about other people; and, perceived-stigma describes a person’s beliefs about negative attitudes that other people hold. Structural stigma describes policies and cultural norms restricting an individual’s resources, opportunities and wellbeing [17].

In the context of suicide, stigma may be associated with a range of experiences including suicide bereavement, attempted suicide, suicide ideation, caring for someone who has attempted suicide or being touched by suicide in another way. Stigma is described as both an outcome of, and a contributing factor to, this broad range of suicide experiences [18]—where an experience of suicide may lead to stigma (of self and by others) and stigma associated with poor social and emotional wellbeing becomes a risk factor for suicide [19]. The consequences of exposure to suicide—whether through bereavement, attempted suicide or suicidal ideation—are wide ranging for individuals and their families, and include judgement, guilt, self-blame, shame, embarrassment and concealment of cause of death [19–21]. Stigma, both perceived and actual, following suicide is also associated with increased risk of suicidal thoughts, suicide attempt, non-suicidal self-harm, depression, heightened psychological distress and a greater need for psychiatric care [13, 22]. The experience of stigma is more likely following suicide bereavement than other sudden deaths by unnatural causes [20].

International research has identified higher levels of self-stigma when seeking help for personal problems in rural populations versus urban populations [23]. However, rural populations are not heterogeneous. For example, the Australian farming context can affect how stigma is experienced, and responded to, as demonstrated by the common cultural mores of ‘being the first to offer help to someone else but the last to ask for help myself’ and ‘by admitting I am struggling people will see me as weak and no longer trust me’ [10]. The Agrarian myths of clean wholesome living, pride, physical strength, ruggedness and self-worth are intertwined with hard work, personal struggle and self-sacrifice [24]. Additionally, conforming to masculinist norms—for example, denouncing emotional openness and vulnerability, favouring self-reliance and encouraging risk-taking [25]—has also been demonstrated to increase self-stigma and result in less positive attitudes towards help-seeking [26, 27]. It is, however, important to draw the distinction between gender and sex—that is that gender does not reside in the person but refers to the socially constructed roles, behaviours, activities, and attributes that society considers appropriate for men and women [28].

In Australia’s rural communities, ‘masculinist’ norms are also displayed by females [10] working and living in agricultural communities, and stigma is exhibited by a broader cross-section of the community. Recent work by Klingelschmidt and colleagues [12] confirms a significant excess risk of suicide among agricultural, forestry and fishing workers with no significant differences between males and females.

In small rural communities—where anonymity is generally low and available mental health services limited—critical contributors to self-stigma include the apprehension of confiding in others and subsequent fear around confidentiality [29]. Informal social support buffers the negative effects of perceived-stigma for people bereaved by suicide. Conversely, poor social support is associated with high levels of perceived-stigma [13] and, according to Courtenay [30], lacking social relationships and support is a risk factor for death—especially for men. In rural farming communities, where social networks are small and tightly entwined, an experience of suicide may place strain on these social connections and further increase the prevalence of stigma.

Australian efforts to measure suicide stigma have generally been applied to a relatively limited range of community samples, such as undergraduate university students and Facebook respondents [31, 32]. Only recently has this focus begun to extend to broader community samples [33]. To date, there has been minimal effort to measure how the rural and farming context may influence the experience of and response to suicide stigma, apart from a small study focusing on rural young people [34].

### The effect of knowledge about suicide on stigma

Evidence suggests that increasing mental health literacy is associated with a reduction in stigma associated with mental health or suicide [33, 35]. Increasing mental health literacy reportedly supports help seeking behaviour, with increased understanding and recognition of signs and symptoms resulting in improved attitudes toward seeking support [36]. However, recent reviews raise questions about the strength of the evidence of reduced mental health stigma through improving mental health literacy [37, 38]. More recently, stigma reduction efforts have become more targeted, with efforts made to increase knowledge and awareness about suicide as a method of reducing suicide stigma and risk, and bolstering suicide prevention efforts [39]. While Australian research has looked at the relationship between suicide stigma and suicide literacy in community samples [32], there has been no specific focus on rural populations or those known to be affected by an experience of suicide.

### The effect of an experience of suicide

Recent research has explored levels of exposure to suicide (knowing someone who died by suicide) and the impact of such exposure on those who are bereaved [40, 41]. Results highlighted that a single suicide death touches many more lives than previously reported. Compounding this, exposure to suicide has been linked with increased levels of depression and anxiety, posttraumatic stress disorder and increased suicide risk [42]. A developing focus is on whether having a kinship relationship with the deceased has particular bearing on this—with current evidence suggesting that relationship closeness (social and emotional closeness), rather than kinship, has a greater bearing on how people are affected by suicide exposure [41]. To date, some Australian research has identified rural and remote communities as particularly vulnerable to suicide exposure and its impact [43]. However, there is a lack of evidence about the effect of suicide exposure within the close social networks and predominantly family-owned farming environment of rural Australia.

In the light of evidence to date, this paper reports on baseline data of an online intervention designed to:

- Identify the effect of an intervention to reduce self-stigma and perceived-stigma experienced by members of Australia’s rural farming community with lived experience of suicide.
- Increase suicide literacy in the farming community and explore the relationship between change in self-stigma and perceived-stigma of suicide, suicide literacy, and the nature of suicide experience, age and health behaviour measures.

## Methods

### Study design

The study was a mixed methods online intervention developed from a strengths-based perspective [44]—considered appropriate given previous research identifying this populations as being goal-directed and avoiding emotional vulnerability [10]—and tailored to the demographics and personalised experience of participants. The quantitative component of the research describes the baseline data from the validated tools, and compares these results to previously published values. The qualitative elements of the research explored participants’ experience of suicide and experience of, and response to, suicide stigma in greater detail. A more detailed explanation of the study methods can be found in the research protocol [45].

The intervention was set over a maximum of 12-weeks (self-paced), with data collected at baseline and post-intervention, as well as during the participants’ involvement. This paper reports on the baseline data collected immediately following participants’ registration on the website. Ethics was provided by Deakin University Human Research Ethics Committee (Ref: 2015–136).

### Sample

The target population was stipulated by *beyondblue*, the funding body (Ref. CLT:7241), to focus on men from the Australian farming community aged 30–64 years who self-identified as affected by an experience of suicide. Experience of suicide included bereaved by suicide, attempted suicide, cared for someone who attempted suicide, had thoughts of taking their own life, or been touched by suicide in some other way. Previous research by *beyondblue* [46] had identified service gaps, specifically:

- Few programs targeting the needs of men aged 30–64 years, compared with younger age groups,
- Reduced digital engagement among those aged over 64 years, and
- Higher levels of perceived-stigma associated with poor mental health for men aged 30–64 years.

Given these identified gaps, recruitment and study design primarily considered men from the farming community with an experience of suicide aged 30–64 years. However, the authors recognised that other members of the rural and farming community beyond the target group were likely to be affected by suicide. Males outside of the target age group (18–30 and over 64 years) and females were, therefore, included in the study. This initial paper reports on baseline data of all participants.

### Recruitment

Recruitment was framed around knowledge that rural farming community members are very willing to offer help to others yet less willing to ask for help themselves [10]. Accordingly, a call to action was made for people to proactively share personal information and insights to help others—assisting to develop ways to further rural suicide prevention and improve support for all those affected. Study recruitment took a broad, ‘snowballing’ approach, engaging a wide range of strategies and utilising rural networks via mainstream and social media, digital and hard copy flyers, website (including [47]), partner agency networks, community presentations, voluntary ‘Community Champions’, and a large stakeholder network kept informed and engaged via online newsletters.

### Tools

Data was collected online, via a website designed to be cognisant of the rural and farming context. The website allowed as easy access as possible for participants isolated both geographically and psychologically. This was achieved by using a digital platform that was optimised for slow internet connection (a continuing concern in many parts of rural Australia) [48], and accessible on a range of digital devices, such as older PCs, smart phones and tablets. Participants were de-identified, using the website anonymously to enable open and meaningful participation. Personalisation of the user experience included language and imagery reflective of participants’ gender and farming type as well as recognition of participants’ specific experience of suicide. For example:

- Male participants from dairy farms saw imagery of male dairy farmers, while female participants from cropping farms would see imagery of female cropping farmers.
- Participants identifying as bereaved by suicide would be delivered information framed within the context of the bereavement experience—‘Following a suicide death, it is not unusual for those left behind to feel a range of conflicting emotions’—while different language was used for participants identifying as carers of someone who had attempted suicide—‘As a carer of someone who attempted to take their own life, it is not unusual to experience feelings of…’.
- To ascertain baseline stigma and literacy, assessment tools were also adapted for delivery in the online environment.

#### Stigma of Suicide Scale (SOSS)

Perceived and self-stigma were measured using an adaption of the short-form Stigma of Suicide Scale (SOSS) [31]. The SOSS has robust psychometric properties and has been validated in Australian community samples of undergraduate student and Facebook samples [31, 32]. The short-form SOSS scale comprises 16 items with three subscales identifying stigma, isolation/depression and normalisation/glorification. Items are assessed using a 5-point Likert scale [49]. An adaption of the introductory wording was made in consultation with the lead author of the SOSS, Associate Professor Philip Batterham, to reflect the changed focus from general suicide stigma (introduced in the original SOSS by the statement ‘In general, people who suicide are…’) to this study’s focus on perceived- and self-stigma of suicide. All participants completed the perceived-stigma SOSS, introduced by the statement ‘In general, other people think that a person who takes their own life is…’ Only participants identifying as having attempted—or had thoughts of attempting—suicide were required to complete the self-stigma SOSS, as introduced by the statement ‘Because I have attempted to take my own life I feel…’ or ‘Because I have had thoughts of taking my own life I feel…’.

#### Literacy of Suicide Scale (LOSS)

Suicide literacy was measured using the Literacy of Suicide Scale (LOSS) [32]. The LOSS has robust psychometric properties and has been validated in an Australian community sample of undergraduate students [32]. The scale comprises 12 true/false items assessing participants’ knowledge about suicide.

#### Effect of suicide

Following establishment of participants’ suicide experience, the study utilised assessment methods previously used in the US by Cerel and colleagues [41, 42] and in Australia by Maple and colleagues [40] to establish participants’ relationship to the person/s who had died, and the effect of bereavement on the participants’ life.

## Results

### Participant profile

**Fig. 1:**
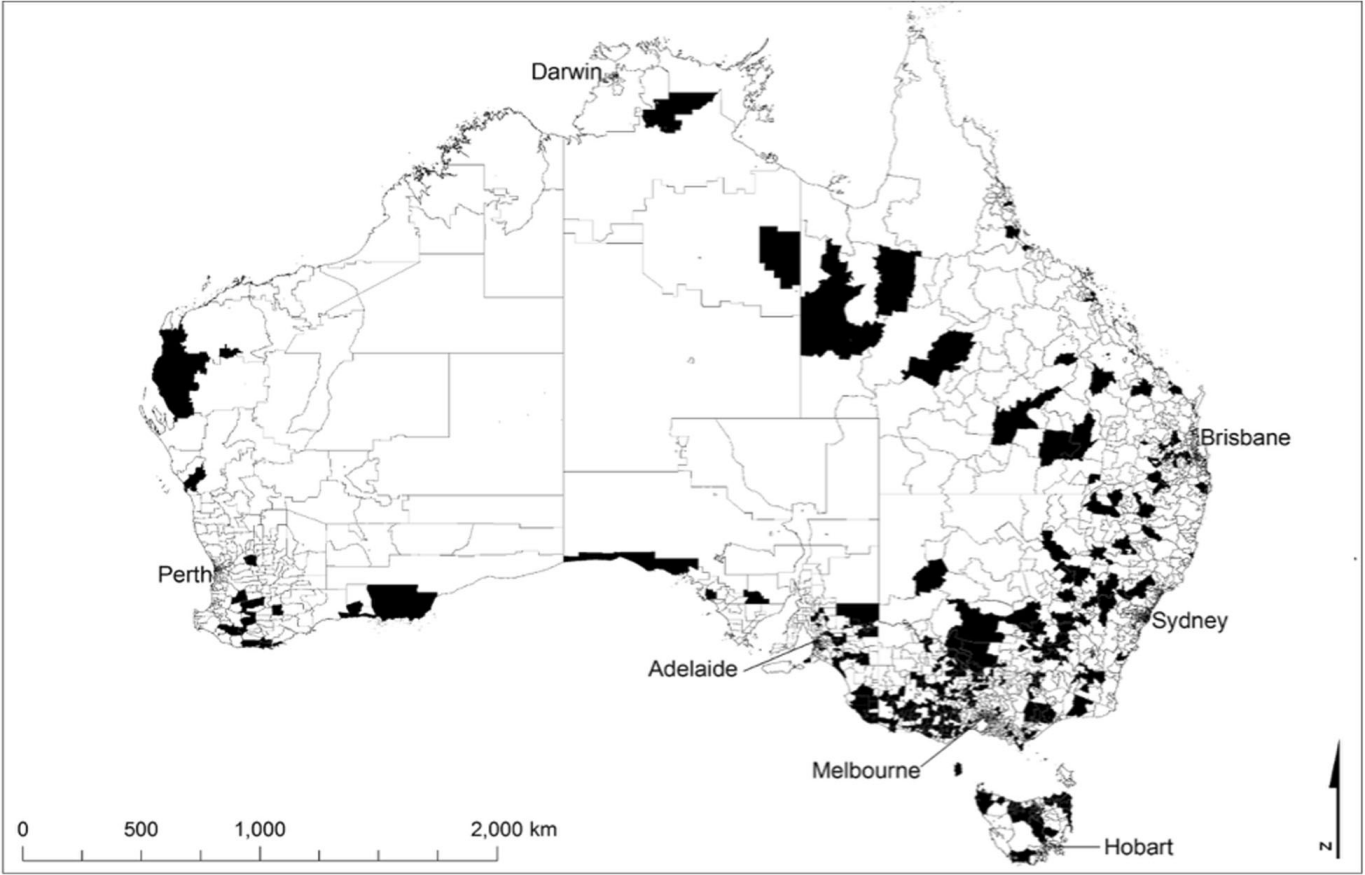
Postcode location of Ripple Effect participants from July 2016–May 2017

Participants engaged from every state and mainland territory across Australia with particular engagement noted in rural and remote areas. There was minimal penetration in capital cities and urban locations as shown in Fig. 1.

Participant demographics (*N* = 536) are presented in Table 1, identifying higher female (*N* = 351, 65.5%) than male participation (*N* = 185, 34.5%), despite the design and marketing focus towards males.

**Table 1:** Demographics of Ripple Effect participants

		Gender^a^		Total participants
		Male	Female	
Age	18–24 years	13 7.0%)	33 (9.4%)	46 (8.6%)
	25–49 years	93 (50.3%)	206 (58.7%)	299 (55.8%)
	50+ years	79 (42.7%)	112 (31.9%)	191 (35.6%)
Total participants		185 (34.5%)	351 (65.5%)	536 (100.0%)

^a^Note: 26 participants were excluded as they either did not specify age (*n* = 22) or listed gender as other (*n* = 4)

**Fig. 2:**
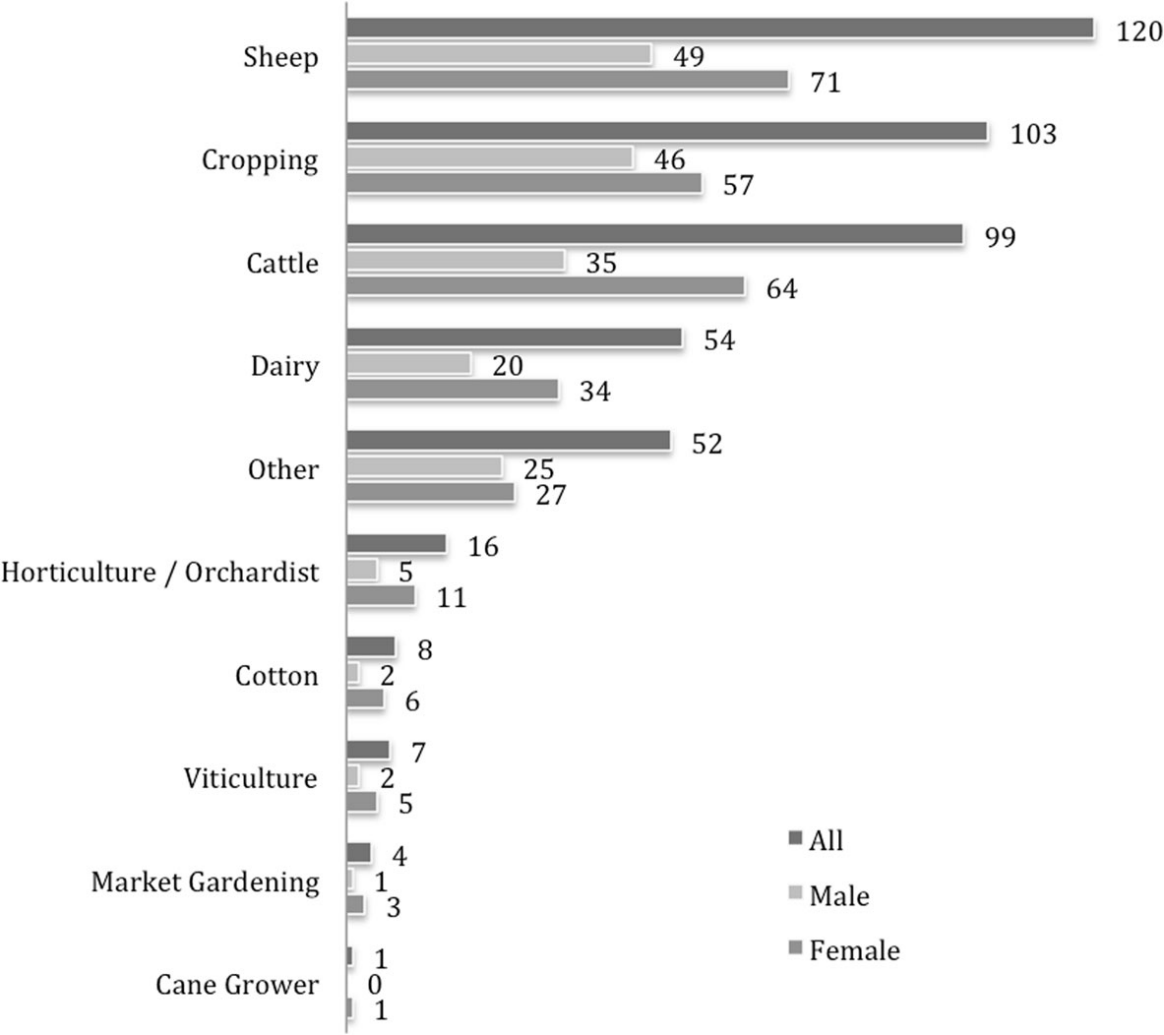
Farming type among participants currently farming (*n* = 276) Note: totals exceed *n* = 276 as participants involved in mixed farming enterprises may have nominated more than one farming type

The participant group represented a broad cross-section of rural Australia, with those involved in farming or having previously farmed comprising the majority of the participants (62%, *n* = 276). Of those actively farming, a diverse range of farming types were represented (see Fig. 2)—suggesting a range of possible stressors and contextual factors influencing exposure to, and effect of, an experience of suicide. Remaining participants reported having never farmed (38%). Data was not collected on whether those ‘no longer farming’ were influenced to leave farming as a result (directly or indirectly) of their experience of suicide.

Digital access to the website by participants was spread across a range of devices including desktop devices (PC/laptop) 51.3%, smart phone 37.3% and tablet 11.4%.

All participants identified as having experience of suicide. Given the website focus, this was expected, and no option was provided for those not identifying as being affected by suicide. People not identifying as having been affected by suicide could not progress beyond this question.

Male participants were significantly more likely to have had a direct personal experience of suicide (attempted or had thoughts of suicide) than females (χ^2^(1), 6.79, *p* = 0.009) (Table 2). This difference was primarily driven by gender differences in thoughts about suicide, with male participants (21.4%) more likely to have considered taking their own life compared with females (12.8%). Gender differences were identified with respect to suicide bereavement, with males (44.8%) less likely to report suicide bereavement than female participants (50.4%), although this was not statistically significant (χ^2^(1), 1.59, *p* = 0.208). When focusing specifically on suicide bereaved participants, males (27.9%) were significantly less likely than females (53.0%) to be bereaved by the loss of a family member (χ^2^(1), 14.88, *p* < 0.001). Further variation was identified in the affect that suicide bereavement had on participants. Males (14.0%) were significantly less likely to have experienced a suicide death as having a continuing significant or devastating outcome than females (33.7%) (χ^2^(1), 11.45, *p* = 0.001).

**Table 2:** Summary of experience of suicide

User Type N (%)	All^b^ (*N* = 562)	Male All (*N* = 192)	Female All (*N* = 359)
I have attempted to take my own life	38 (6.8)	14 (**7.3**)	22 (**6.1**)
I have been touched by suicide in some other way	109 (21.2)	40 (20.8)	78 (21.7)
I have cared for someone who attempted to take their own life	44 (7.8)	11 (5.7)	32 (8.9)
I have had thoughts about taking my own life	88 (15.7)	41 (**21.4**)	46 (**12.8**)
Someone I know took their own life	268 (47.7)	86 (44.8*)*	181 (50.4*)*
Someone I know took their own life	**N** **= 268**	**N** **= 86**	**N** **= 181**
*Was this person a family member (Yes/No)*			
No response (participants have not yet completed question)^a^	22 (8.2)	6 (7.0)	16 (8.8)
Yes	120 (21.4)	24 (27.9)	96 (53.0)
No	126 (22.4)	56 (65.1)	69 (38.1)
Effect on your life	**N = 268**	**N = 86**	**N = 181**
No response^a^	70 (26.1)	22 (25.6)	47 (26.0)
The death had a significant or devastating effect on me that I still feel	73 (27.2)	12 (14.0)	61 (33.7)
The death disrupted my life in a significant or devastating way, but I no longer feel that way	31 (11.6)	7 (8.1)	24 (13.6)
The death disrupted my life for a short time	36 (13.4)	14 (16.3)	22 (12.1)
The death had somewhat of an effect on me, but did not disrupt my life	49 (18.2)	26 (30.2)	23 (12.7)
The death had little effect on my life	9 (3.3)	5 (5.8)	4 (2.2)

^a^The high rate of no response reflects the fact that only people who identified as being bereaved by suicide were presented with this question. In addition, not all participants had yet reached this stage in their personalised website pathway in order to be able to complete the question)

^b^4 participants marked ‘other’ and 7 participants did not provide gender information

Bold entries highlight points discussed in the text or represent summed totals of a number of categories

Gender variation was seen among participants bereaved by suicide relative to levels of closeness with the person who had died (see Table 3). Of those participants who identified as bereaved by suicide and responded to the question of closeness to the deceased (*n* = 184), 35.4% of females described being close or very close to the deceased compared to 23.3% of males (χ^2^(1), 4.26, *p* = 0.039).

**Table 3:** Suicide bereaved participants closeness to deceased

	Gender		
	Male N(%)	Female N(%)	Total participants N(%)
Very close	**11 (12.8)**	**42 (23.2)**	**53 (19.9)**
Close	**9 (10.5)**	**22 (12.2)**	**31 (11.6)**
Moderately close	14 (16.3)	23 (12.7)	37 (13.9)
A bit close	9 (10.5)	15 (8.3)	24 (9.0)
Not close	15 (17.4)	24 (13.3)	39 (14.6)
No response^a^	28 (32.6)	55 (30.4)	83 (31.1)
Total	86 (100)	181 (100)	267 (100)

^a^High rates of no response due to the fact that not all participants had yet reached this stage in their personalised website pathway in order to be able to complete the question

Bold entries highlight points discussed in the text or represent summed totals of a number of categories

#### Suicide stigma

Although no statistical comparison can be made (given the difference in introductory wording to the SOSS reflecting self- and perceived-stigma), participants indicated a higher level of stigma, lower levels of attributing suicide to isolation/depression and lower levels of normalisation/glorification than the community sample (see Table 4). Reliability of the adapted SOSS (as measured by Cronbach’s alpha) was generally identified as good to excellent with only two results considered questionable [50].

**Table 4:** Stigma comparison between Ripple Effect sample and Community Sample [32]

	*n* (%)	SOSS – Stigma mean (SD) [α]	SOSS – Isolation/depression mean (SD) [α]	SOSS – Glorification/normalisation mean (SD) [α]
Ripple Effect self-stigma (total completing baseline self-SOSS)	**98**	**2.79 (0.91) [0.890]**	**3.67 (1.09) [0.911]**	**2.05 (0.83) [0.795]**
*Age group*				
18–24	13	3.13 (1.05) [0.91]	3.92 (1.02) [0.84]	1.94 (0.95) [0.88]
25–49	58	2.83 (0.88) [0.88]	3.72 (1.07) [0.91]	1.99 (0.78) [0.75]
50+	24	2.54 (0.85) [0.87]	3.50 (1.13) [0.92]	2.20 (0.85) [0.81]
*Gender*				
Male	46	2.73 (0.95) [0.91]	3.60 (1.11) [0.90]	2.24 (0.86) [0.79]
Female	49	2.82 (0.90) [0.89]	3.71 (1.11) [0.93]	1.87 (0.78) [0.81]
Ripple Effect perceived-stigma group (total completing baseline perceived-SOSS)	**420**	**2.86 (0.84) [0.90]**	**3.71 (0.71) [0.80]**	**2.00 (0.64) [0.73]**
*Age group*				
18–24	40	2.95 (0.91) [0.92]	3.84 (0.64) [0.88]	1.98 (0.76) [0.85]
25–49	217	2.88 (0.85) [0.90]	3.78 (0.69) [0.81]	1.96 (0.60) [0.69]
50+	148	2.82 (0.82) [0.90]	3.59 (0.68) [0.71]	2.07 (0.65) [0.73]
*Gender*				
Male	148	2.90 (0.82) [0.90]	3.63 (0.73) [0.83]	2.02 (0.64) [0.83]
Female	267	2.82 (0.86) [0.90]	3.74 (0.70) [0.78]	2.01 (0.64) [0.63]
Community Sample (total sample completing SOSS)	**1405 (100.0)**	**2.19 (0.83) [0.89]**	**4.11 (0.83) [0.72]**	**2.45 (0.82) [0.82]**
*Age group*				
18–24	438 (31.2)	2.26 (0.82)	4.19 (0.70)	2.44 (0.79)
25–49	394 (28.0)	2.15 (0.84)	4.12 (0.85)	2.46 (0.83
50+	491 (34.9)	2.13 (0.81)	4.07 (0.88)	2.43 (0.85)
*Gender*				
Male	553 (39.4)	2.35 (0.85)	4.04 (0.82)	2.50 (0.87)
Female	767 (54.6)	2.05 (0.77)	4.18 (0.81)	2.39 (0.78)

Bold entries highlight points discussed in the text or represent summed totals of a number of categories

Within the participant group, comparing the differences in subscales for self-stigma between gender, the only significant result was for the Glorification/Normalisation subscale (*t* = − 2.20 (93), *p* = 0.030). There were no differences across the three subscales when gender was compared for perceived stigma.

#### Suicide literacy

Participants had a significantly higher level of suicide literacy than a previous Australian community sample undertaken by Batterham and Callear [32], both overall and when comparing gender (all comparisons *p* < 0.001) (see Table 5). Statistically significant differences across all age groups (all comparisons p < 0.001) were evident when compared with the community sample. Within the participant group, females scored significantly higher than males on the LOSS (*t* = 2.17 (428), *p* = 0.031). No significant differences in LOSS scores between the age groups were identified in this sample.

**Table 5:** Suicide literacy comparison between Ripple Effect sample and Community Sample (online) [32]

	n (%)	LOSS – Literacy Mean (SD)
Ripple Effect Sample (total sample completing the baseline LOSS)	**435 (100.0)**	**10.1 (1.47)**
*Age group* ^*a*^		
18–24	42	10.07 (1.44)
25–49	227	10.17 (1.57)
50+	150	10.09 (1.26)
*Gender* ^*a*^		
Male	152	9.91 (1.50)
Female	278	10.23 (1.44)
Community Sample (online)	**1405 (100.0)**	**7.65 (2.47)**
*Age group*		
18–24	438 (31.2)	8.20 (2.17)
25–49	394 (28.0)	7.90 (2.32)
50+	491 (34.9)	7.01 (2.68)
*Gender*		
Male	553 (39.4)	7.36 (2.59)
Female	767 (54.6)	7.88 (2.35)

^a^Only participants specifying gender/age were included in the age group and gender baseline measurement of suicide literacy

Bold entries highlight points discussed in the text or represent summed totals of a number of categories

## Discussion

### Patterns of study engagement

Australia’s rural farming community demonstrated considerable national engagement within the study period, as reflected by the location of participants from across the country, providing support for the appropriateness of recruitment and community engagement strategies. Wide ranging access is encouraging, given that metropolitan Australia continues to have higher internet access (88%) than remote and very remote Australia (79%) [51], with quality of connectivity varying considerably.

Patterns of accessibility of the study website (51.3% PC/laptop, 37.3% smart phone and 11.4% tablet) varied from patterns of internet access previously measured in rural Australia—41.1% PC/laptop, 34.4% smart phone and 24.6% tablet [51]. This may reflect website content and the level of engagement involved, with people allocating a block of time to participate in in-depth and emotional content—most likely in the privacy of their own home—rather than opportunistically accessing online data such as the weather or social media.

Whilst designed for males aged 30–64 years, younger and older male participants and females also participated. This is not unexpected. Younger Australians demonstrate familiarity and established engagement with digital and online technology [51]. Young adult participation has implications for future development of online suicide-stigma reduction interventions, with a requirement for tailored content beyond that already contained on the study website. High female participation was also not unexpected, given previous evidence of the dominance of female participation in suicide research in Australia and internationally [41]. It is also pertinent given the significant excess risk of suicide among agricultural, forestry and fishery workers in both females and males [12]. Additionally, the importance of females’ roles in ensuring the wellbeing of their families is well recognised [52], particularly in farming [53], and the prevalence of females may indirectly result in information transfer and stigma reduction for rural men. Anecdotal evidence shared with the study team indicated that participating females actively encouraged male family members and friends to participate.

### The effect of varying experience of suicide

Explorations of participants’ suicide experience identified a greater proportion of females as bereaved by the suicide death of a family member. This is not unexpected given the higher rates of male suicide [54], translating to more females likely to have lost male family members to suicide. Female’s greater likelihood of experiencing a devastating and ongoing impact of suicide bereavement may reflect that females are more likely to have lost family members to suicide than target males. Given the nature of family farming, this may mean the loss of key labour and knowledge in the farming business—in addition to the loss of a relationship—with women having to compensate for the loss of husband/brother/father/son/co-manager of farming duties. The greater impact on bereaved females may also be a reflection of their closeness to the deceased. While previous research suggests that level of closeness has greater bearing on the affect of suicide bereavement than kinship [43], results from this study suggest kinship may have particular bearing for this population—as affected by the prevailing nature of family owned farms [55]—where family life and work are tightly entwined [56] and a loss by suicide is likely to have a significant impact.

As a whole, the study sample reported considerably lower ongoing significant or devastating effect of suicide bereavement, when compared with a recent online survey of the general Australian population [43]. Of those bereaved by suicide, 37% were affected in this way (compared with 27% in the current study). This difference may be due to the pragmatic response to suicide bereavement identified among people in Australian farming communities—where a practical, action-oriented focus takes precedence over one based on emotion and there is a de-sensitisation to death [10]. It is also likely to be a factor of reported lower levels of closeness to the deceased when compared to the general Australian population study—31.4% of the current study sample self-reported as ‘very close’ or ‘close’ to the deceased, compared with 50% in the general Australian population sample. This is consistent with research linking the effect of suicide bereavement with level of closeness to the deceased [41, 42].

Higher proportions of males identifying as having attempted or considered suicide, when compared with females, is counter to existing research which estimates approximately 64% of all hospital admissions for self-inflicted injury are female and suggests Australian women are significantly more likely to have thoughts about suicide, make a suicide plan or make a suicide attempt than men [57, 58]. However, these figures acknowledge the difficulty in accurately estimating gender differences in suicide attempts due to non-reporting of attempts that do not result in hospitalisation, unclear intent behind injuries and subsequent likely under reporting, and inability to separate self-harm without suicide attempt from an attempt to take one’s life. Given the higher rates of farmers identifying as males [59], higher rates of male suicide in rural areas [60] and that the Ripple Effect was promoted as a suicide prevention tool for farming communities, it is understandable that the project may have appealed more to males who had considered or attempted suicide and rural females concerned about males in their lives. It may also be that the anonymity and non-judgemental environment of the Ripple Effect facilitated greater willingness by men to openly share emotional vulnerability without fear of sanction.

This research highlights the need to avoid a concept of masculinity that homogenises experience and behaviour for males (and females) without considering the unique geographic, psychological and social contexts that individuals experience, and which necessarily influence life, social connection and work in rural farming communities [61]. Male participant’s greater likelihood of having attempted suicide or had thoughts of suicide can be understood from within Hogan and colleagues [27] gendered framework, suggesting male farmers loss of a coherent agrarian self (tough men in a tough rural environment [62]) and lack of a continued social practice can lead to a ruptured identity. This loss of self—combined with a sense of shame and a contextually driven reluctance to acknowledge (or avoid) problems—can contribute to the will to suicide [27]. This is particularly notable in a population where patriarchy is strong, and men continue to have greater access to power and economic resources. While farming men may be at heightened risk of suicide and stigma, addressing these risks within a framework that recognises contextually influenced patterns of masculinity shows that stigma reduction is possible.

The current results, identifying no difference between women and men in attempted suicide, are pertinent and reflect the recent systemic review undertaken by Klingelschmidt and colleagues [12] which identified no difference in excess of suicide risk among females and males employed in agriculture, fishing and forestry. It will also be interesting to note whether differences will be found through varied patterns and outcomes of engagement during participants’ progression through the intervention.

Males were less likely to have a significant or devastating outcome following suicide bereavement, when compared with female participants. This is consistent with previous research on suicide impact and closeness identifying females as more likely to have poorer outcomes—including suicide ideation, depression, anxiety, posttraumatic stress disorder and prolonged grief—when reporting high impact suicide exposure [42]. This lack of significant or devastating outcome also fits with the masculinist rural norms of toughness in the face of difficult times, self-reliance and avoidance of help seeking. It will be interesting to explore the qualitative data—shared as part of the intervention—by participants identifying as having experienced a significant or devastating outcome following suicide bereavement, relative to these potential negative outcomes.

### The relationship between suicide literacy and stigma

High participant suicide literacy levels were not reflected by reduced levels of stigma when compared with Batterham and colleague’s community sample [32]. This raises a number of points relative to the relationship between literacy and stigma requiring further examination:

- Is it valuable and/or meaningful to compare heterogeneous populations on measures of suicide literacy and stigma, when the context in which this occurs can be diverse?
- Recent questioning of evidence supporting the positive link between mental health literacy and stigma reduction suggests that more exploration is required in the context of suicide literacy and stigma [37, 38].
- An increasing body of research—in other population groups and occupations—has identified concomitant high literacy levels and high levels of stigma [63, 64]. Building suicide literacy—while often considered best practice [33]—may, therefore, not be an effective means of reducing suicide stigma, particularly in populations where high levels of literacy are already evident.

### Strengths and limitations

This study attracted considerable participation (Fig. 1) from rural farming communities across rural Australia, with a diversity of farming involvement and geographical location, and a wide range of suicide experience. However, study participants were self-selected and not necessarily representative, given the heterogeneity of Australia’s rural community. Given the requirement for participants to self-identify some experience of suicide, the meaningfulness of comparison to a general community sample was limited.

Study design and delivery drew on the National Centre for Farmer Health’s many years of experience engaging—and developing trust—with the rural farming community. This was supported by a number of partner agencies and community members and ensured broad coverage and recruitment, despite the sensitive nature of the research.

Online study delivery limited accessibility to participants with internet connectivity, a particular concern for remote Australia where online access is less likely [51]. Efforts were made to encourage participation in the project by those without internet access, but were unable to include the collection of baseline assessments of stigma and literacy. Efforts were also made to design online content in a responsive format, allowing access to content at varying degrees of resolution dependent on quality of internet connection. Despite these challenges, online study methods provided a geographically and experientially diverse study sample, which may not have been possible using other methods.

The findings of this study have limited generalisability to other populations, although population groups internationally have been identified as facing similar challenges to Australian farming communities (for example, farming communities in North America). Further limits to generalisability arise due to the adaption of the SOSS to measure self- and perceived-stigma. This prohibits meaningful comparisons with research using the original general measure of suicide stigma [31]. The study design, however, does have potential to be translatable and transferable, following the tailoring of content to other population groups.

## Conclusion

This paper reported on the baseline data from a rural farming community population with high levels of suicide literacy. Results from this study challenge several previous findings of research relative to suicide experience, gendered experience, stigma and literacy. This suggests the need for greater understanding of the relationship between stigma, literacy and suicide experience within the psychological, geographical and cultural context of rural work and life. Further research is required, to investigate how ‘best practice’ can be developed and delivered to effectively reduce stigma, support help seeking and assist suicide prevention efforts within rural farming populations.

## Abbreviations

LOSS: Literacy of Suicide Scale
NCFH: National Centre for Farmer Health
SOSS: Stigma of Suicide Scale

## References

[1] Hirsch JK, Cukrowicz CZ (2014). Suicide in rural areas: an updated review of the literature. Journal of Rural Mental Health.

[2] Milner A, Niven H, LaMontagne AD. Occupational class differences in suicide: evidence of changes over time and during the global financial crisis in Australia. BMC Psychiatry. 2015;15(223) 10.1186/s12888-015-0608-5 PMC4578370 26391772

[3] Kennedy A, Maple MJ, McKay K, Brumby SA. Suicide and accidental death in Australia’s rural farming communities: a review of the literature. Rural Remote Health. 2014:2517. 24909931

[4] Hanigan I, Butler C, Kokic P, Hutchinson M (2012). Suicide and drought in new South Wales, Australia, 1970–2007. Proc Natl Acad Sci.

[5] Andersen K, Hawgood J, Klieve H, Kolves K, De Leo D (2010). Suicide in selected occupations in Queensland: evidence from the state suicide register. Aust N Z J Psychiatry.

[6] Miller K, Burns C (2008). Suicides on farms in South Australia, 1997–2001. Aust J Rural Health.

[7] Ramadas S, Kuttichira P (2017). Farmers’ suicide and mental disorders perspectives in research approaches: comparison between India and Australia. International Journal of Community Medicine and Public Health.

[8] Caldwell TM, Jorm AF, Dear KB (2004). Suicide and mental health in rural, remote and metropolitan areas in Australia. Med J Aust.

[9] Perceval M, Kolves K, Reddy P, De Leo D (2017). Farmer suicides: a qualitative study from Australia. Occup Med.

[10] Kennedy AJ. Life, death and the experience of suicide and accidental death bereavement for Australia's rural farming families: University of New England; 2016.

[11] Albrecht G (2007). Solastalgia: the distress caused by environmental change. Australasian Psychiatry.

[12] Klingelschmidt J, Milner A, Khireddine-Medouni I, Witt K, Alexopolous EC, Toivanen S, LaMontagne AD, Chastang JF, Niedhammer I (2018). Suicide among agricultural, forestry, and fishery workers: a systematic literature and meta-analysis. Scand J Work Environ Health.

[13] Pitman A, Rantell K, Marston L, King M, Osborn D. Perceived stigma of sudden bereavement as a risk factor for suicidal thoughts and suicide attempt: analysis of British cross-sectional survey data on 3387 young bereaved adults. Int J Environ Res Public Health. 2017;14(286) 10.3390/ijerph14030286 PMC5369122 28282958

[14] Butler S, editor. The Macquarie dictionary. Sydney: Macquarie dictionary: Publishers; 2013.

[15] Merriam-Webster Dictionary [https://www.merriam-webster.com/dictionary/stigma].

[16] The stigma of Ment Illn is making us sicker: Why Ment Illn should be a Public Health priority [https://www.psychologytoday.com/blog/brick-brick/201405/the-stigma-mental-illness-is-making-us-sicker].

[17] *beyondblue*: Information Paper: Stigma and discrimination associated with depression and anxiety In*.* Hawthorn: *beyondblue*; 2015.

[18] Oexle N, Rusch N. Stigma - risk factor and consequence of suicidal behavior : implications for suicide prevention. Nervenarzt. 2017; Epub ahead of print 10.1007/s00115-017-0450-8 29147725

[19] Carpiniello B, Pinna F. The Reciprocal Relationship between Suicidality and Stigma. Frontiers in Psychiatry. 2017, 8(35) 10.3389/fpsyt.2017.00035 PMC5340774 28337154

[20] Pitman AL, Osborn DPJ, Rantell K, King MB (2016). The stigma perceived by people bereaved by suicide and other sudden deaths: a cross-sectional UK study of 3432 bereaved adults. J Psychosom Res.

[21] Maple M, Cerel J, Jordan J, McKay K (2014). Uncovering and identifying the missing voices in suicide bereavement. Suicidology Online.

[22] Scocco P, Preti A, Totaro S, Ferrari A, Toffol E (2017). Stigma and psychological distress in suicide survivors. J Psychosom Res.

[23] Stewart H, Jameson JP, Curtin L (2015). The relationship between stigma and self-reported willingness to use mental health services among rural and urban older adults. Psychol Serv.

[24] Bryant L, Garnham B (2015). The fallen hero: masculinity, shame and farmer suicide in Australia. Gender, Place and Culture.

[25] Brannon R, David D, Brannon R (1976). The male sex role: Our culture's blueprint for manhood, and what it's done for us lately. The forty-nine percent majority: The male sex role.

[26] Wasylkiw L, Clairo J. Help seeking in men: when masculinity and self-compassion collide. Psychology of Men and Masculinity. 2016;

[27] Hogan A, Scarr E, Lockie S, Chant B (2012). Ruptured identity of male farmers: subjective crisis and the risk of suicide. Journal of Rural Social Sciences.

[28] Gender Fact Sheet [http://www.who.int/en/news-room/fact-sheets/detail/gender].

[29] Clement S, Schauman O, Graham T, Maggioni F, Evans-Lacko S, MBezborodovs N, Morgan CD, Rusch N, Brown JS, Thornicroft G (2015). What is the impact of mental health-related stigma on help-seeking? A systematic review of quantitative and qualitative studies. Psychol Med.

[30] Courtenay WH, Courtenay WH (2010). Rural Men’s health: situating Men’s risk in the negotiation of masculinity. Dying to be men: psychosocial, environmental, and biobehavioral directions in promoting the health of men and boys.

[31] Batterham P, Callear A, Christensen H (2013). The stigma of suicide scale: psychometric properties and correlates of the stigma of suicide. Crisis.

[32] Batterham P, Callear A, Christensen H (2013). Correlates of suicide stigma and suicide literacy in the community. Suicide Life Threat Behav.

[33] Peel R, Buckby B, McBain KA. Comparing the effect of stigma on the recognition of suicide risk in others between Australia and Brazil *GSTF*. J Psychol. 2017;3(2)

[34] Bartik W, Maple M, McKay K (2015). Suicide bereavement and stigma for young people in rural Australia: a mixed methods study. Advances in Mental Health.

[35] Gulliver A, Griffiths KM, Christensen H, Mackinnon A, Calear AL, Parsons A, MBennett K, Batterham PJ, Stanimirovic R (2012). Internet-based interventions to promote mental health help-seeking in elite athletes: an exploratory randomized controlled trial. J Med Internet Res.

[36] Reavley NJ, Jorm AF (2012). Public recognition of mental disorders and beliefs about treatment: changes in Australia over 16 years. Br J Psychiatry.

[37] Stuart H (2016). Reducing the stigma of mental illness. Global Mental Health.

[38] Gronholm P, Henderson C, Deb T, Thornicroft G (2017). Interventions to reduce discrimination and stigma: the state of the art. Soc Psychiatry Psychiatr Epidemiol.

[39] Gullestrop J, Lequertier B, Martin G (2011). MATES in construction: impact of a multimodal, community-based program for suicide prevention in the construction industry. Int J Environ Res Public Health.

[40] Maple M, Cerel J, Sanford R, Pearce T, Jordan J (2017). Is exposure to suicide beyond kin associated with risk for suicidal behavior? A systematic review of the evidence. Suicide Life Threat Behav.

[41] Cerel J, Maple M, Aldrich R, van de Venne J (2013). Exposure to suicide and identification as survivor: results from a random-digit dial survey. Crisis.

[42] Cerel J, Maple M, van de Venne J, Brown M, Moore M, Flaherty C (2017). Suicide exposure in the population: perceptions of impact and closeness. Suicide Life Threat Behav.

[43] Maple M, Kwan M, Borrowdale K, Riley J, Murray S, Sanford R (2016). The Ripple Effect: Understanding the Exposure and Impact of Suicide in Australia. In*.* Sydney: Suicide Prevention Australia.

[44] Hammond W. Principles of strength-based practice. In*.* Calgary, Alberta: resiliency. Initiatives. 2010;

[45] Kennedy AJ, Versace VL, Brumby SA (2016). Research protocol for a digital intervention to reduce stigma among males with a personal experience of suicide in the Australian farming community. BMC Public Health.

[46] *beyondblue*: Unpublished data from http://www.beyondblue.org.au, shared with the research team In*.*; 2013.

[47] Farmer Health website [http://www.farmerhealth.org.au/].

[48] Park S, Freeman JL, Middleton C, Allen M, Eckermann R, Everson R (2015). The multi-layers of digital exclusion in rural Australia. *48th Hawaii International Conference on System Sciences*.

[49] Likert R (1932). A technique for the measurement of attitudes. Archives of Psychology.

[50] George D, Mallery P (2003). SPSS for windows step by step: a simple guide andreference. 11.0 update.

[51] Household use of information technology, Australia, 2014–15 [http://www.abs.gov.au/ausstats/abs@.nsf/mf/8146.0].

[52] Alston M (2012). Rural male suicide in Australia. Soc Sci Med.

[53] Brumby S (2013). Farm Work and Family Health: A Study on Farming Family Heath across selected Agricultural Industries in Australia.

[54] Facts and stats about suicide in Australia [http://www.mindframe-media.info/for-media/reporting-suicide/facts-and-stats].

[55] Clark N, O'Callaghan P (2013). Australian farming families are here to stay. Farm Policy Journal.

[56] Brumby S, Willder S, Martin J (2010). Milking their health for all its worth? Improving the health of farming families through facilitated learning. Extension Farming Systems Journal.

[57] McKenna K, Harrison JE (2012). Hospital separations due to injury and poisoning, Australia 2008–09.

[58] Harrison JE, Henley G (2014). Suicide and hospitalised self-harm in Australia: trends and analysis.

[59] Agricultural Commodities, Australia, 2016–17 [http://www.abs.gov.au/AUSSTATS/abs@.nsf/allprimarymainfeatures/97B95C93A7FD9B75CA2573FE00162CAF?opendocument].

[60] Australian Bureau of Statistics: Suicides, Australia, 2010. In*.*; 2012.

[61] Connell RW, Messerschmidt JW (2005). Hegemonic masculinity: rethinking the concept. Gend Soc.

[62] Connell R, Campbell H, Bell MM, Finney M (2006). Country/City Men. In: Country boys: Masculinity and Rural Life edn.

[63] Edwards JL, Crisp DA (2016). Seeking help for psychological distress: barriers for mental health professionals. Aust J Psychol.

[64] Oliffe JL, Ogrodniczuk JS, Gordon SJ, Creighton G, Kelly MT, Black N, Mackenzie C (2016). Stigma in male depression and suicide: a Canadian sex comparison study. Community Ment Health J.

